# Nottingham Prognostic Index Plus: Validation of a clinical decision making tool in breast cancer in an independent series

**DOI:** 10.1002/cjp2.32

**Published:** 2016-01-15

**Authors:** Andrew R Green, Daniele Soria, Jacqueline Stephen, Desmond G Powe, Christopher C Nolan, Ian Kunkler, Jeremy Thomas, Gillian R Kerr, Wilma Jack, David Cameron, Tammy Piper, Graham R Ball, Jonathan M Garibaldi, Emad A Rakha, John MS Bartlett, Ian O Ellis

**Affiliations:** ^1^ Division of Cancer and Stem CellsBreast Cancer Pathology Research Group, School of Medicine, University of Nottingham, Nottingham City HospitalHucknall RoadNottinghamNG5 1PB; ^2^ Intelligent Modelling & Analysis Research GroupSchool of Computer ScienceUniversity of Nottingham, Jubilee CampusWollaton RoadNottinghamNG8 1BB; ^3^ Advanced Data Analysis Centre, University of Nottingham, University ParkNottinghamNG7 2RD; ^4^ School of Molecular, Genetic and Population Health SciencesCentre for Population Health Sciences, Medical School, University of EdinburghTeviot PlaceEdinburghEH8 9AG; ^5^ Cellular Pathology, Nottingham University Hospitals NHS TrustHucknall RoadNottinghamNG5 1PB; ^6^ The Institute of Genetics and Molecular MedicineEdinburgh Cancer Research Centre, University of Edinburgh, Western General HospitalCrewe Road SouthEdinburghEH4 2XR; ^7^ Edinburgh Breast Unit, Western General HospitalCrewe Road SouthEdinburghEH4 2XU; ^8^ John van Geest Cancer Research Centre, School of Science and Technology, Nottingham Trent UniversityNottinghamNG11 8NS; ^9^ Transformative PathologyOntario Institute for Cancer Research, MaRS Centre661 University Avenue, Suite 510TorontoCanadaM5G 0A3

**Keywords:** breast cancer, classification, prognostic index, molecular, clinical, outcome

## Abstract

The Nottingham Prognostic Index Plus (NPI+) is a clinical decision making tool in breast cancer (BC) that aims to provide improved patient outcome stratification superior to the traditional NPI. This study aimed to validate the NPI+ in an independent series of BC. Eight hundred and eighty five primary early stage BC cases from Edinburgh were semi‐quantitatively assessed for 10 biomarkers [Estrogen Receptor (ER), Progesterone Receptor (PgR), cytokeratin (CK) 5/6, CK7/8, epidermal growth factor receptor (EGFR), HER2, HER3, HER4, p53, and Mucin 1] using immunohistochemistry and classified into biological classes by fuzzy logic‐derived algorithms previously developed in the Nottingham series. Subsequently, NPI+ Prognostic Groups (PGs) were assigned for each class using bespoke NPI‐like formulae, previously developed in each NPI+ biological class of the Nottingham series, utilising clinicopathological parameters: number of positive nodes, pathological tumour size, stage, tubule formation, nuclear pleomorphism and mitotic counts. Biological classes and PGs were compared between the Edinburgh and Nottingham series using Cramer's V and their role in patient outcome prediction using Kaplan–Meier curves and tested using Log Rank. The NPI+ biomarker panel classified the Edinburgh series into seven biological classes similar to the Nottingham series (*p* > 0.01). The biological classes were significantly associated with patient outcome (*p* < 0.001). PGs were comparable in predicting patient outcome between series in Luminal A, Basal p53 altered, HER2+/ER+ tumours (*p* > 0.01). The good PGs were similarly validated in Luminal B, Basal p53 normal, HER2+/ER− tumours and the poor PG in the Luminal N class (*p* > 0.01). Due to small patient numbers assigned to the remaining PGs, Luminal N, Luminal B, Basal p53 normal and HER2+/ER− classes could not be validated. This study demonstrates the reproducibility of NPI+ and confirmed its prognostic value in an independent cohort of primary BC. Further validation in large randomised controlled trial material is warranted.

## Introduction

Breast cancer (BC), is one of the leading causes of death in women but it represents a very heterogeneous group of tumours in terms of genotype, phenotype, behaviour and response to treatment. With the number of available treatment options, making the most appropriate treatment choice is increasingly difficult and complex and, therefore, a more personalised management of BC is required. However, clinical decision making in personalised BC treatment requires robust and accurate risk stratification based on outcome prediction and biology 1.

Personalised treatment plans for BC require integration of clinical, histopathological and biological information to effectively stratify patients with regard to their expected outcome and response to the various applicable treatment options. There has been increasing interest in use of multigene assays, such as Oncotype DX^®^ 2 and MammaPrint^®^ 3, and their potential clinical utility in BC management. However, the incorporation of molecular taxonomy of BC using gene expression profiling into routine clinical decision‐making has not proved entirely successful due to factors including reproducibility, validation, cost and lack of utility for all BC patients.

The current Nottingham Prognostic Index (NPI) 4, 5, 6 is based on a combination of histopathological factors (tumour size, lymph node stage and tumour grading) integrated in a prognostic index formula 7 which can be used to stratify BC patients with operable early stage primary BC into prognostic groups. The utility of the NPI has been confirmed after long‐term follow‐up 4, validated independently in large multi‐centre studies 5, 8, 9, revised to stratify patients into additional prognostic groups 10, and is currently adopted in clinical practice in the UK and other parts of Europe and Australia. However, the NPI does not reflect the biological heterogeneity of BC and assigns equal weighting of the prognostic factors histological grade, tumour lymph node stage and tumour size to all cancers. It, therefore, requires further enhancement to support more accurate personalised management of BC patients.

It is now recognised that the biological characteristics of BC are important for clinical management and addition of biological markers to the NPI can significantly improve risk stratification of BC patients 11. We have, therefore, developed the Nottingham Prognostic Index Plus (NPI+) 11 which conceptually evolved to modernise the historical NPI by applying the prognostic methods used in the NPI, which are based on well‐established powerful clinicopathological variables, following BC molecular class assignment. NPI+ is thus based on a two tier evaluation; the initial assessment determines the biological class of the tumour (detailed below) and is subsequently followed by classification using traditional clinicopathological prognostic variables using a tailored (bespoke) NPI‐like prognostic formulae for each biological class 11, 12, 13, 14. NPI+ uses routine clinical samples and commonplace laboratory methods and could integrate easily into current international clinical practice. It has potential clinical utility by providing improved patient outcome stratification and by providing a decision making tool which can identify patients likely to have a good outcome following conventional BC treatment and a subgroup(s) of patients at risk of adverse outcome, that is, who are at increased risk of treatment failure and who could potentially benefit from additional/alternative therapy, should these currently be available or become available in the future 11. Seven core BC NPI+ Biological Classes are initially determined by the evaluation of 10 BC‐related biomarkers using immunohistochemistry and a fuzzy rule induction algorithm 15 to classify the breast tumours. The molecular classes identified based on the combination of these 10 biomarkers using fuzzy logic are similar in biomarker profile to those intrinsic classes identified using gene expression profiling and include three luminal classes (Luminal A, N and B), two basal classes (Basal – p53 altered and Basal – p53 normal) and two HER2+ classes (HER2+/ER+ and HER2+/ER−) 15. These distinct biological classes of BC showed significant association with patient outcome 12, 13, 14. Each NPI+ Biological Class is subsequently stratified using a set of well‐defined prognostic clinicopathological variables which are combined in bespoke formulae to stratify each individual NPI+ Biological Class into two or more prognostic subgroups (NPI+ Prognostic Groups) which have been been shown to be superior to the classic NPI 11.

In this study, we aimed to validate the NPI+ in a large independent series of clinically annotated early stage BCs from a single centre (Edinburgh, UK) to assess the potential of NPI+ as a prognostic tool in BC.

## Materials and methods

### Nottingham series

A series of 1073 patients from the Nottingham‐Tenovus Primary Breast Carcinoma Series, aged 70 years or less, presenting with primary operable (stages I, II and IIIa) invasive BC between 1986 and 1998 were previously used to develop the NPI+ 11, 12, 13, 14. This is a well‐characterised consecutive series of patients who were uniformly treated according to locally agreed clinical protocols 12, 16. All tumours were less than 5‐cm diameter on clinical/pre‐operative measurement and/or on operative histology (T1 and T2). Women aged over 70 years were not included because of the increased confounding factor of death from other causes and because primary treatment protocols for these patients often differed from those for younger women. Adjuvant systemic therapies were offered according to the NPI 2 and hormone receptor (HR) status 2, 10. Patients in the Moderate I group (NPI 3.41–4.4) with HR‐positive tumours were offered hormonal therapy. Patients in the Moderate II (NPI 4.41–5.4) and Poor (NPI ≥ 5.41) groups received hormone therapy for HR‐positive tumours and cytotoxic therapy [classical cyclophosphamide, methotrexate and 5‐fluorouracil (CMF)] for HR‐negative tumours if the patient was fit enough to tolerate chemotherapy. Hormonal therapy was given to 396 patients (40.3%) and chemotherapy to 192 (18.9%). A total of 19 patients (1.9%) in the Moderate II or Poor prognostic groups received a combination of chemotherapy and endocrine therapy (Table 1). Data relating to survival were collated in a prospective manner for those patients presenting after 1989 only.

**Table 1:** Pathological characteristics of the Nottingham and Edinburgh series

	Nottingham (*n* = 1073) *n* (%)	Edinburgh (*n* = 885) *n* (%)	*p*‐value
**Grade**			
1	158 (14.7)	194 (22.0)	
2	348 (32.4)	359 (40.8)	<0.001
3	567 (52.8)	327 (37.2)	
**Tubule formation**			
1	53 (5.0)	73 (8.3)	
2	346 (33.0)	222 (25.3)	0.002
3	651 (62.0)	582 (66.4)	
**Pleomorphism**			
1	19 (1.8)	3 (0.3)	
2	378 (36.1)	346 (39.5)	0.121
3	651 (62.1)	528 (60.2)	
**Mitosis**			
1	349 (33.2)	523 (59.6)	
2	190 (18.1)	138 (15.7)	<0.001
3	511 (47.6)	216 (24.6)	
**Size**			
	0.13–10 cm (median 2.0 cm)	0.4–7.0 cm (median 1.7 cm)	
<1.5 cm	240 (22.4)	285 (33.6)	<0.001
≥1.5 cm	833 (77.6)	564 (66.4)	
**Stage**			
1	654 (61.0)	614 (69.4)	
2	330 (30.8)	211 (23.8)	0.014
3	88 (8.2)	60 (6.8)	
**Nottingham prognostic index**			
Excellent	110 (10.3)	137 (15.5)	
Good	200 (18.6)	251 (28.4)	
Moderate 1	293 (27.3)	248 (28.0)	
Moderate 2	277 (25.8)	178 (20.1)	<0.001
Poor	140 (13.0)	53 (6.0)	
Very poor	45 (4.2)	17 (1.9)	
**Treatment**			
None	410 (40.3)	95 (10.7)	
Chemotherapy	192 (18.9)	118 (13.3)	<0.001
Endocrine therapy	396 (38.9)	581 (65.6)	
Chemotherapy/endocrine therapy	19 (1.9)	91 (10.3)	
**Survival**			
	0.4–25.7 years (median 14.3 years)	0.2–25.5 years (median 11.4 years)	
Alive	582 (54.2)	584 (66.0)	
BC‐specific deaths	328 (30.1)	179 (20.2)	<0.001
Non‐BC‐related deaths or lost to follow‐up	163 (15.2)	122 (13.8)	

### Edinburgh series

The Edinburgh series comprised a cohort of 885 patients treated by breast conservation surgery, axillary node sampling or clearance and whole breast radiotherapy between 1981 and 1998 in Edinburgh (Edinburgh Breast Conservation Series) 17. Patients were those considered suitable for breast‐conserving therapy and were T1 or T2, N0 or N1 and M0 for conventional tumour node metastasis staging. Patients with larger primary tumours and those with multi‐focal cancers on preoperative assessment were not considered eligible for inclusion. Standard surgical treatment was wide local excision. Patients with tumours measuring >2 cm in diameter and/or clinically N1 received a Level III axillary clearance. For tumours measuring clinically ≤2 cm, a lower axillary node sample (minimum four nodes) was undertaken. Post‐operative breast radiotherapy was given at a dose of 45 Gy in 20 daily fractions in patients with one or more pathologically involved node on an axillary node sample; the peripheral lymphatics were also irradiated over 4 weeks. Patients received adjuvant systemic therapy as follows: endocrine therapy (primarily using tamoxifen), chemotherapy alone (primarily using CMF), chemotherapy plus endocrine therapy or no adjuvant systemic therapy (primarily those with grade 1 tumours).

Breast cancer‐specific survival (BCSS) is defined as the interval between the operation and death from BC, death being scored as an event, and patients who died from other causes or were still alive were censored at the time of last follow‐up.

This study was approved by the Nottingham Research Ethics Committee 2 under the title Development of a molecular genetic classification of breast cancer'.

### Determination of NPI+ biological class

Immunohistochemical reactivity for the NPI+ biomarkers in the Nottingham series was previously determined using standard immunohistochemical techniques on tumour samples prepared as tissue microarrays (TMAs) 12. TMAs for both cohorts were prepared using 0.6‐mm cores. For the Nottingham series, one TMA core from the centre and one from the periphery of the most representative areas of tumour were included 12. For the Edinburgh series, one TMA core per patient from representative tumour areas was used 18. The NPI+ was developed using the following biomarkers: Estrogen Receptor (ER), Progesterone Receptor (PgR), cytokeratin (CK) 5/6, CK7/8 (using the anti‐cytokeratin CAM5.2 clone), epidermal growth factor receptor (EGFR; HER1), c‐erbB2 (HER2), c‐erbB3 (HER3), c‐erbB4 (HER4), p53 and Mucin 1 14. TMAs of the Edinburgh series were also stained for these same biomarkers in Nottingham using the same procedures as previously described (Supporting Information Table 1) 12, 14. A series of BC, prepared as TMAs, with differing levels of expression of the 10 biomarkers (ranging from negative to strongly positive) were included as positive and negative controls and to standardise immunoreactivity. Levels of immunohistochemical reactivity were determined by microscopic analysis using the modified Histochemical score (H‐score), giving a semi‐quantitative assessment of both the intensity of staining (0–3) and the percentage of positive cells (0–100) (multiplied to give values between 0 and 300) 19, 20. Immunohistochemical staining and subsequent scoring, conducted by at least two independent scorers, was performed in the Nottingham laboratory. For HER2, the American Society of Clinical Oncology/College of American Pathologists Guidelines Recommendations for HER2 Testing in BC were used for assessment 21. In the Nottingham series, equivocal (2+) HER2+ cases were confirmed by chromogenic *in situ* hybridisation as previously described 22. The Reporting Recommendations for Tumour Marker Prognostic Studies (REMARK) criteria, recommended by 23, were followed. In the Edinburgh series, equivocal cases (*n* = 67) were excluded from analysis.

For biological classification, a fuzzy logic rule‐based method algorithm was used where the cut‐offs for each biomarker were previously determined 15. In particular, the median value of markers was used for ER, PgR, CK7/8, HER3, HER4 and MUC1. The expertise values were used for those markers that had a median equal to zero and for those where clinicians were sure about the value to consider (CK5/6, EGFR, p53 and HER2). Pathological characteristics of the 885 cases, along with the Nottingham cases, are summarised in Table 1. Hormonal therapy was given to 581 patients (65.6%), chemotherapy to 118 (13.3%) and 91 patients (10.3%) received a combination of chemotherapy and endocrine therapy (Table 1).

### Determination of NPI+ prognostic groups

The NPI+ Prognostic Groups were then calculated using bespoke NPI‐like formulae, previously developed in each NPI+ Biological Class of the Nottingham series, utilising the existing available clinicopathological parameters (Table 2) 11. Briefly, these were established by utilisation of the Beta values generated by Cox regression analysis in predicting BCSS of the well‐established histopathologic prognostic factors. These formulae were initially derived from the Biological Classes in Green *et al* 2013 14 and were subsequently refined using the improved biological classification used in Soria *et al* 2013 15 consisting of: number of positive nodes, nodal ratio, pathological tumour size, stage, tubule formation, nuclear pleomorphism and mitotic counts. These were identified as the most significant variables in the Nottingham series impacting on survival, according to their Beta value in Cox regression indicating the magnitude of the influence of the hazard. The Nottingham series was split into the NPI+ Biological Classes and Cox regression analyses were performed independently for each class to identify the most significant clinicopathological prognostic factors and their beta value in the context of the individual classes. NPI+ Prognostic Groups for the Edinburgh series were assigned using the categorical cutpoints previously derived from the Nottingham series in each of the NPI+ Biological Classes 11. For this purpose, the original pathology assessments on full‐face sections for the histopathological parameters were utilised.

**Table 2:** NPI+ formulae for the biological classes

Class	NPI+ formula
Luminal A	(0.8 × Mitosis) + (0.5 x Size) + (1.8 × Nodal ratio*)
Luminal N	(0.8 × Tubules) + (0.6 × Stage)
Luminal B	(0.7 × Mitosis) + (1.0 × Nodal ratio)
Basal p53 altered	(1.4 × Nodal ratio) + (0.4 × Size)
Basal p53 normal	(0.6 × Stage) + (1.8 × Pleomorphism)
HER2+/ER+	(0.5 × Size) + (0.9 × Stage)
HER2+/ER−	(0.9 × Stage) − (0.6 × Nodal ratio)

* Number of nodes positive/Total number of nodes.

### Statistical analysis

The association between NPI+ Biological Classes and both histopathological and clinical characteristics was assessed using Cramer's V 24. BCSS between NPI+ Biological classes and NPI+ Prognostic Groups was determined using Kaplan–Meier curves and tested using Log Rank. A *p* < 0.01 was considered significant with Bonferroni adjustment for multiple testing.

## Results

### Clinicopathological parameters of the Edinburgh series

There were significant differences in the distribution of grade and size (both *p* < 0.001) of the breast tumours between the Nottingham and Edinburgh series with a larger proportion of the Nottingham series being of larger tumour size, and of higher grade and stage (Table 1). The median follow‐up for the Nottingham series was 14.3 years and the Edinburgh series was 11.4 years. A total of 328 (36.0%) and 179 (20.2%) patients died due to their disease in the Nottingham and Edinburgh series, respectively. The Edinburgh series had better BCSS (82.1%) over the first 10‐year period compared with the Nottingham series (74.7%).

### NPI+ biological class

**Figure 1:**
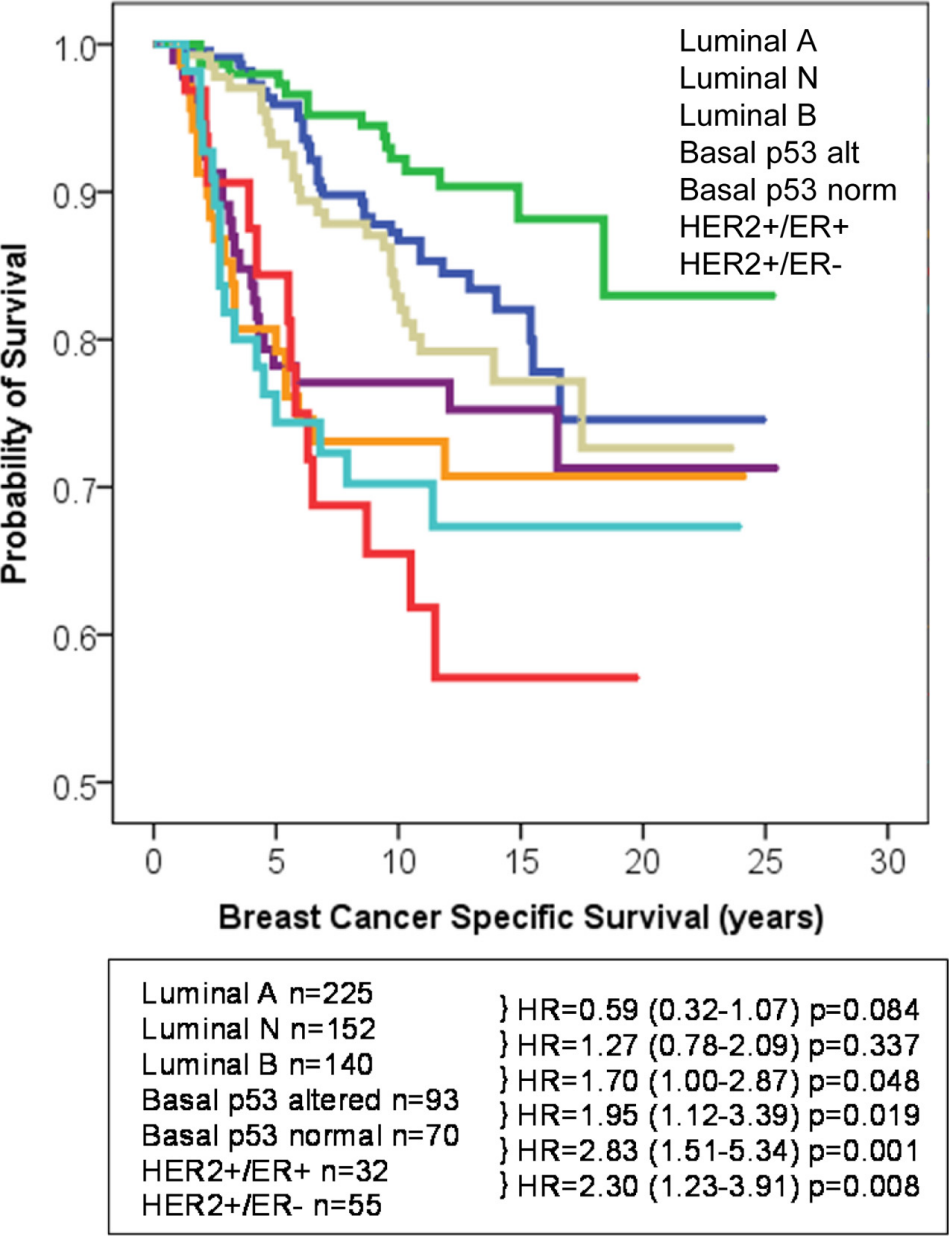
BCSS of the Edinburgh series with respect to NPI+ Biological Classes.

NPI+ Biological Class was determined in the Edinburgh series using the immunohistochemical data for the 10 NPI+ Biomarkers: this showed that there was a similar distribution between each of the seven NPI+ Biological Classes (Luminal A, Luminal N, Luminal B, Basal p53 altered, Basal p53 normal, HER2+/ER+ and HER2+/ER−) compared with the Nottingham series (*p* = 0.629, Table 3). A total of 51 cases (5.8%) were not assigned to any class compared with 3.5% in the Nottingham series. There were significant associations between the clinicopathological parameters of the Edinburgh series and the NPI+ Biological Classes which are summarised in Table 4. The NPI+ Biological Classes were significantly associated with patient survival where the Luminal and Basal classes had a better BCSS than the HER2+ classes (Figure 1).

**Table 3:** Distribution of NPI+ biological classes within the Nottingham and Edinburgh series

NPI+ Class	Nottingham (*n* = 1073)*n* (%)	Edinburgh (*n* = 885)*n* (%)	*p*‐value
Luminal A	288 (26.8)	225 (29.3)	
Luminal N	205 (19.1)	152 (19.8)	
Luminal B	186 (17.3)	140 (18.3)	
Basal p53 altered	113 (10.5)	93 (12.1)	*p* = 0.629
Basal p53 normal	96 (8.9)	70 (9.1)	
HER2+/ER+	62 (5.8)	32 (3.6)	
HER2+/ER−	85 (7.9)	55 (6.2)	
Unclassified	38 (3.5)	51 (5.8)	

**Table 4:** Clinicopathological parameters of the NPI+ BC biological classes in the Edinburgh series

	Luminal A (*n* = 219) *n* (%)	Luminal N (*n* = 144) *n* (%)	Luminal B (*n* = 137) *n* (%)	Basal – p53 altered (*n* = 90) *n* (%)	Basal – p53 normal (*n* = 64) *n* (%)	HER2+/ER+ (*n* = 32) *n* (%)	HER2+/ER− (*n* = 55) *n* (%)	Cramer's V (*p*‐value)
**Size**								
<15 mm	89 (40.6)	67 (46.5)	50 (36.5)	12 (13.3)	18 (28.1)	7 (23.3)	11 (21.2)	0.169 (<0.001)
≥15 mm	130 (59.4)	77 (53.5)	87 (63.5)	78 (86.7)	46 (71.9)	23 (76.7)	41 (78.8)	
**Grade**								
1	75 (33.6)	47 (30.9)	35 (25.0)	1 (1.1)	3 (4.4)	3 (9.7)	1 (1.8)	
2	108 (48.4)	73 (48.0)	72 (51.4)	10 (10.8)	23 (33.8)	8 (25.8)	23 (41.8)	0.381 (<0.001)
3	40 (17.9)	32 (21.1)	33 (23.6)	82 (88.2)	42 (61.8)	20 (64.5)	31 (56.4)	
**Lymph node stage**								
1	172 (76.4)	108 (71.1)	100 (71.4)	68 (73.1)	50 (71.4)	15 (46.9)	31 (56.4)	
2	44 (19.6)	40 (26.3)	36 (25.7)	18 (19.4)	18 (25.7)	10 (31.2)	15 (27.3)	0.169 (<0.001)
3	9 (4.0)	4 (2.6)	4 (2.9)	7 (7.5)	2 (2.9)	7 (21.9)	9 (16.4)	
**NPI**								
Excellent	55 (24.8)	33 (21.9)	23 (16.4)	0	1 (1.5)	2 (6.2)	0	
Good	82 (36.9)	54 (35.8)	53 (37.9)	5 (5.4)	15 (22.1)	5 (15.6)	11 (20.0)	
Moderate 1	52 (23.4)	34 (22.5)	37 (26.4)	39 (41.9)	29 (42.6)	7 (21.9)	17 (30.9)	
Moderate 2	22 (9.9)	26 (17.2)	21 (15.0)	37 (39.8)	19 (27.9)	10 (31.2)	16 (29.1)	0.221 (<0.001)
Poor	11 (5.0)	4 (2.6)	4 (2.9)	8 (8.6)	4 (5.9)	6 (18.8)	8 (14.5)	
Very poor	0	0	2 (1.4)	4 (4.3)	0	2 (6.2)	3 (5.5)	
**Adjuvant therapy**								
Chemotherapy	5 (2.2)	12 (7.9)	4 (2.9)	25 (26.9)	21 (30.0)	6 (18.8)	16 (29.1)	
Hormone therapy	185 (82.2)	100 (65.8)	112 (80.0)	42 (45.2)	32 (45.7)	19 (59.4)	26 (47.3)	
Hormone therapy/chemotherapy	14 (6.2)	21 (13.8)	11 (7.9)	10 (10.8)	6 (8.6)	4 (12.5)	6 (10.9)	0.230 (<0.001)
No therapy	21 (9.3)	19 (12.5)	13 (9.3)	16 (17.2)	11 (15.7)	3 (9.4)	7 (12.7)	

### NPI+ prognostic groups

There were a similar number of NPI+ Prognostic Groups evident in each of the biological classes in the Edinburgh series compared with the Nottingham series, however, there was a significant difference in the distribution of the NPI+ Prognostic Groups between the Nottingham and Edinburgh series (Table 5, *p* < 0.001) 11. Some of the poor NPI+ Prognostic Groups were under‐represented in the Edinburgh series due to the relatively lower frequency of highly proliferative tumours in the series (Table 1) which may also explain the better survival of this series.

**Table 5:** Distribution of the NPI+ Groups in the Nottingham and Edinburgh series

NPI+ group	Nottingham *n* (%)	Edinburgh *n* (%)
**Luminal A**		
1.1	148 (17.9)	160 (21.2)
1.2	83 (10.0)	53 (7.0)
1.3	25 (3.0)	9 (1.2)
**Luminal N**		
2.1	133 (16.1)	151 (20.0)
2.2	17 (2.1)	1 (0.1)
**Luminal B**		
3.1	77 (9.3)	133 (17.6)
3.2	58 (7.0)	3 (0.4)
**Basal – p53 altered**		
4.1	86 (10.4)	78 (10.3)
4.2	10 (1.2)	13 (1.7)
**Basal – p53 normal**		
5.1	44 (5.3)	2 (0.2)
5.2	28 (3.4)	68 (9.0)
**HER2+/ER+**		
6.1	31 (3.7)	15 (1.8)
6.2	25 (3.0)	17 (2.1)
**HER2+/ER−**		
7.1	55 (6.6)	53 (6.4)
7.2	8 (1.0)	0

**Figure 2:**
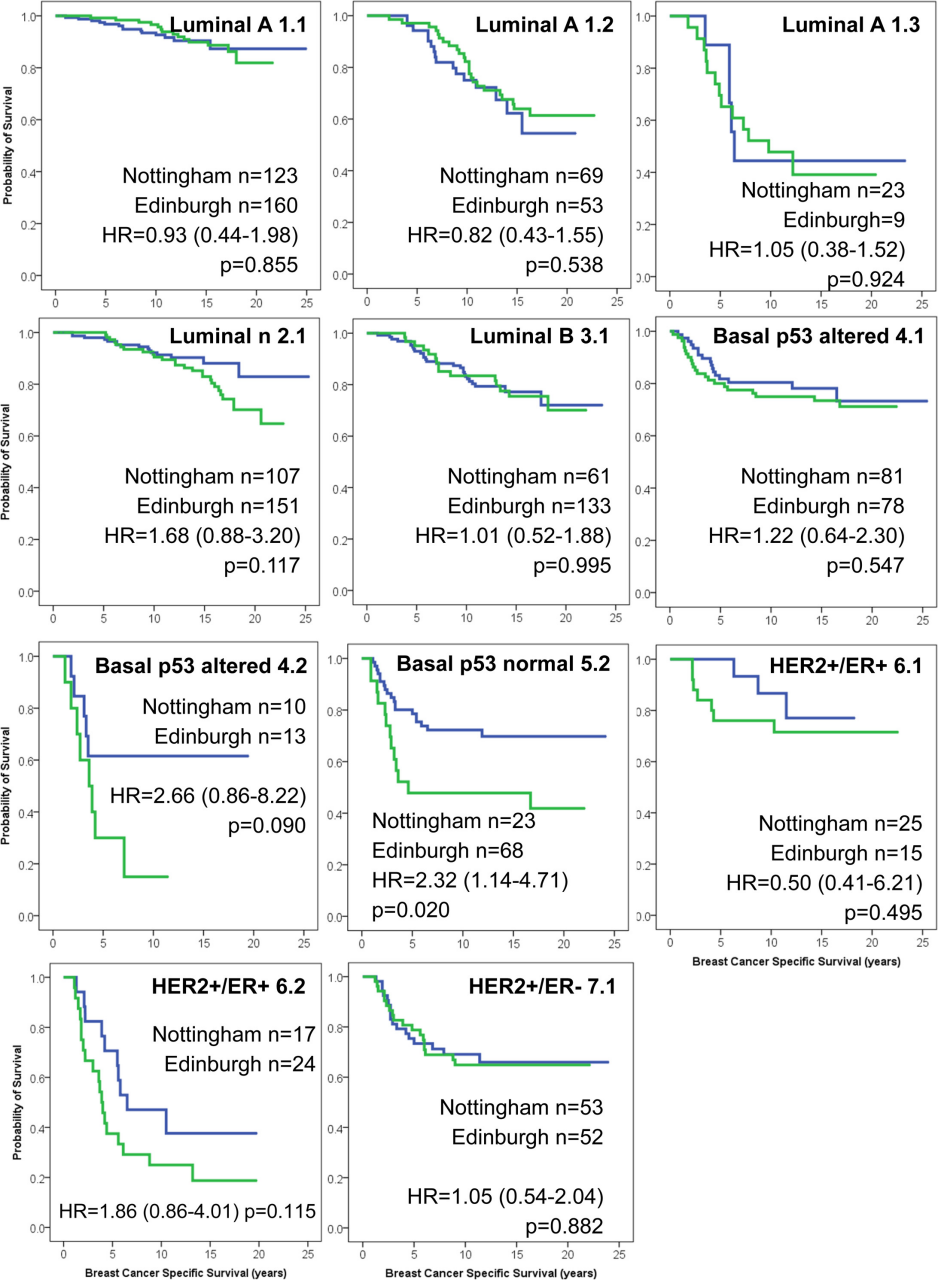
Patient outcome for the NPI+ Prognostic Groups, comparing the Nottingham and Edinburgh series.

Comparison of the BCSS in each of the NPI+ Prognostic Groups between the Nottingham and Edinburgh Series showed there were no significant differences in patient outcome in the majority of NPI+ Prognostic Groups (Figure 2). Luminal A tumours, which had good representation in all three NPI+ Prognostic Groups, showed comparable patient outcome between the Edinburgh and Nottingham Series, as did the BCSS of the Basal p53 altered and HER2+/ER+ tumours. Certain NPI+ Groups (Luminal N Group 1; Luminal B Group 2; Basal p53 normal Group 2; HER2+/ER− Group) could not be compared due to being under‐represented in the Edinburgh series.

## Discussion

We have developed the NPI+ methodology with a view to increasing the information available to clinicians and patients to allow them to offer more personalised choices of adjuvant therapy in all early stage forms of BC. NPI+ was developed on a series of over 1000 BC cases from a single centre (Nottingham, UK) with long‐term follow‐up 11, 12, 13, 14. We have previously demonstrated proof‐of‐principle evidence of its clinical relevance 11, 12, 13, 14. We have, therefore, sought to validate and confirm the prognostic capabilities of NPI+ in a large independent series of BC from a separate centre (Edinburgh, UK).

Although there was some difference in the overall distribution of size, stage and grade of tumours between the Nottingham and Edinburgh series, the distribution of the NPI+ Biological Classes (Luminal A, Luminal N, Luminal B, Basal p53 altered, Basal p53 normal, HER2+/ER+, HER2+/ER−) was similar. This is consistent with the proportion of cancer subtypes reported in other studies 3, 12, 14, 25, 26, 27, 28, 29, 30, 31, 32 and provides evidence that the classification of BC into seven biological classes using a discrete panel of 10 proteins assessed by immunohistochemistry is similar between series.

The second evaluation phase of NPI+ uses well‐established powerful clinicopathological variables to stratify each of the NPI+ Biological Classes into clinically distinct subgroups (NPI+ Prognostic Groups) using bespoke NPI‐like formulae. In all classes, a patient group with a better long‐term outcome was identified which would align with clinical expectation after use of appropriate adjuvant therapy. In the Nottingham series in all seven classes, one or more subgroups of patients were identified who had an adverse long‐term outcome. These latter group(s) of patients are potential candidates for additional/alternative forms of therapy as conventional BC management has failed to mitigate against higher than expected risk of tumour relapse and death from BC. It is envisaged that NPI+ can stratify patients with BCs of any biological class type into a category of expected good outcome following conventional therapy, or one or more categories of adverse outcome following conventional therapy. We fully appreciate that the NPI+ has been developed and validated on archival breast material from patients treated historically in routine practice with either chemotherapy and/or hormone therapy and does not include more contemporary treatments such as trastuzumab. Further validation of NPI+ in key BC randomised clinical trials will allow the prediction of disease recurrence under these certain treatment options.

In the Edinburgh series, the NPI+ Prognostic Groups showed comparable BCSS in the Edinburgh series when compared with the Nottingham series in NPI+ Biological Classes: Luminal A, Basal p53 altered and HER2+/ER+. The NPI+ Prognostic Groups with a better outcome were similarly validated in the NPI+ Biological Classes: Luminal B, Basal p53 normal and HER2+/ER− along with the poor NPI+ Prognostic Group in the Luminal N class. However, due to very small numbers of patients assigned in the Edinburgh series, the remaining NPI+ Prognostic Groups of biological classes Luminal N, Luminal B, Basal p53 normal and HER2+/ER− could not be validated.

In conclusion, this study shows that the distribution of the NPI+ Biological Classes are similar in an independent series of primary BC and we can conclude that biological class determination using the NPI+ biomarker methodology is robust between patient series. We observed similar patterns of patient outcome in the majority of NPI+ Prognostic Groups between the Nottingham and Edinburgh series and can conclude that NPI+ prognostic classification for these groups (all groups of classes Luminal A, Basal p53 altered and HER2+/ER+, the good NPI+ Prognostic Groups of classes Luminal B, Basal p53 normal and HER2+/ER− and the poor NPI+ Prognostic Group of the Luminal N class) appears reproducible. Three of the poor prognostic groups (Luminal N, Luminal B, Basal p53 normal and HER2+/ER−) were under‐represented in the Edinburgh series due to a lower frequency of higher grade tumours and could not be validated in this study.

## Author contributions

ARG, DGP, GRB, JMG, EAR and IOE conceived the study; ARG, CCN and DGP carried out experiments; ARG, DS, JS and DGP performed data analysis; IK, JT, GK, WJ, DC, TP and JMSB provided TMAs together with clinicopathological and outcome data for Edinburgh cases. All authors were involved in data interpretation, writing the paper and did final approval of the submitted and published manuscript.

## Supporting information

Additional Supporting Information may be found in the online version of this article.
The following Supporting information may be found in the online version of this article:

Table 1. Antibodies used in the NPI+.
