# Supplementary material for: Nottingham Prognostic Index Plus: Validation of a clinical decision making tool in breast cancer in an independent series
Source: J Pathol Clin Res. 2016 Jan 15;2(1):32–40. doi: 10.1002/cjp2.32 (PMC4858129; doi:10.1002/cjp2.32)
Supplement: Supplementary file 1 — Table 1. Antibodies used in the NPI+. [file CJP2-2-32-s001.pdf]

Supplementary Table 1. Antibodies used in the NPI+

Antibody	Supplier	Clone	Isotype	Dilution	Antigen Retrieval
Ck5/6	Dako	DS/1684	IgG1	1:50	EDTA
Ck7/8	BD Biosciences	CAM5.2	IgG2a	1:1	Citrate
EGFR	Invitrogen/Zymed	31G7	IgG1	1:30	Proteinase K
ER	Dako	SP1	IgG	1:150	Citrate
HER2	Dako	Rabbit polyclonal	N/A	1:400	None
HER3	Leica	RTJ1	IgM	1:30	Citrate
HER4	Thermo Shandon	Rabbit polyclonal	N/A	1:100	Citrate
MUC1	Leica	NCL-MUC-1	IgG1	1:750	Citrate
p53	Leica	NCL-p53-D07	IgG2b	1:50	Citrate
PgR	Dako	PgR636	IgG1	1:125	Citrate
